# Tracking the circulating SARS-CoV-2 variant of concern in South Africa using wastewater-based epidemiology

**DOI:** 10.1038/s41598-022-05110-4

**Published:** 2022-01-21

**Authors:** Rabia Johnson, Jyoti R. Sharma, Pritika Ramharack, Noluxabiso Mangwana, Craig Kinnear, Amsha Viraragavan, Brigitte Glanzmann, Johan Louw, Nada Abdelatif, Tarylee Reddy, Swastika Surujlal-Naicker, Sizwe Nkambule, Nomfundo Mahlangeni, Candice Webster, Mongezi Mdhluli, Glenda Gray, Angela Mathee, Wolfgang Preiser, Christo Muller, Renee Street

**Affiliations:** 1 Biomedical Research and Innovation Platform (BRIP), South African Medical Research Council (SAMRC), Tygerberg, 7505 South Africa; 2 Division of Medical Physiology, Faculty of Medicine and Health Sciences, Centre for Cardiometabolic Research in Africa, Stellenbosch University, Stellenbosch, South Africa; 3 Pharmaceutical Sciences, School of Health Sciences, University of KwaZulu-Natal, Westville Campus, Durban, 4001 South Africa; 4 Genomics Centre, South African Medical Research Council (SAMRC), Tygerberg, 7505 South Africa; 5 Division of Molecular Biology and Human Genetics, Faculty of Medicine and Health Sciences, DSI-NRF Centre of Excellence for Biomedical Tuberculosis Research, South African Medical Research Council Centre for Tuberculosis Research, Stellenbosch University, Cape Town, South Africa; 6 Biostatistics Research Unit, South African Medical Research Council (SAMRC), Durban, South Africa; 7 Scientific Services, Water and Sanitation Department, City of Cape Town Metropolitan Municipality, Cape Town, South Africa; 8 Environment and Health Research Unit, South African Medical Research Council (SAMRC), Durban, South Africa; 9 Environment and Health Research Unit, South African Medical Research Council (SAMRC), Johannesburg, South Africa; 10 Office of the President, South African Medical Research Council, Tygerberg, 7050 South Africa; 11 Division of Medical Virology at NHLS Tygerberg Hospital and Faculty of Medicine and Health Sciences, Stellenbosch University, Cape Town, South Africa

**Keywords:** RNA, Viral infection, Genotype

## Abstract

This study uses wastewater-based epidemiology (WBE) to rapidly and, through targeted surveillance, track the geographical distribution of SARS-CoV-2 variants of concern (Alpha, Beta and Delta) within 24 wastewater treatment plants (WWTPs) in the Western Cape of South Africa. Information obtained was used to identify the circulating variant of concern (VOC) within a population and retrospectively trace when the predominant variant was introduced. Genotyping analysis of SARS-CoV-2 showed that 50% of wastewater samples harbored signature mutations linked to the Beta variant before the third wave, with the Delta variant absent within the population. Over time, the prevalence of the beta variant decreased steadily. The onset of the third wave resulted in the Delta variant becoming the predominant variant, with a 100% prevalence supporting the theory that the Delta variant was driving the third wave. In silico molecular docking analysis showed that the signature mutations of the Delta variant increased binding to host proteins, suggesting a possible molecular mechanism that increased viral infectivity of the Delta variant.

## Introduction

Since December 2019, the deadly severe acute respiratory syndrome coronavirus-2 (SARS-CoV-2) has caused social and economic disruption and has had a major impact on public healthcare systems globally. In an attempt to control the pandemic, COVID-19 vaccines have been developed at an extraordinary pace. Vaccination programs have become vital due to the emergence of immune-evading Variants of Concern (VOCs) as a result of high mutation rates within the SARS-CoV-2 genome. In this context, a VOC can be classified as a variant of which there is evidence of an increase transmissibility, disease severity and decrease vaccine-induced protection^1^.

Through the course of the pandemic, several rapidly spreading SARS-CoV-2 VOCs have arisen in countries, including the United Kingdom (UK)^2^, South Africa (SA)^3^, Brazil (BZ)^4^, India^5^ and the United States of America (USA)^5–8^. These VOCs include B.1.1.7 (Alpha), B.1.351 (Beta), P.1 (Gamma), B.1.617 (Kappa/Delta) and, more recently, C.1.2. lineage^6,9,10^. The identified lineages are termed VOCs depending on the propensity to infect and transmit due to the acquisition of multiple signature mutations in the viral spike (S) glycoprotein, a key target for vaccine development, potentially affecting viral infectivity and antigenicity^6,11^. The S- protein is a 180–200 kDa protein that is found as a glycosylated trimer surrounding the virion surface. Each monomeric structure consists of an S1 and S2 subunit, which is further characterized by an N-terminal domain (NTD), a receptor-binding domain (RBD), a fusion peptide (FP), heptapeptide repeat sequences 1 and 2 (HR1/2), a transmembrane domain and a cytoplasmic domain^12^. During viral infection, the RBD domains of the trimeric structure confer an “open state” confirmation, which initiates entry into host cells by binding to angiotensin-converting enzyme 2 (ACE 2)^13^. Once bound, the S1 and S2 subunits of the protein are subjected to proteolysis by host proteases. The S1/S2 boundary site residues proline-arginine-arginine-alanine (PRRA) are cleaved by furin convertase, detaching the two subunits, while transmembrane protein 2 (TMPRSS2) cleaves the S2 site (residues KPSK) to expose the fusion peptide. During this time, the HR1/2 peptides undergo conformational modifications to form a “6-helix bundle fusion core” complex that leads to the linking and fusion of the viral and host cell membranes. Once fusion occurs, the viral RNA is released into the host cell for further replication^14,15^. Given the necessity of the spike protein in SARS-CoV-2 replication through viral binding, it is of no surprise that the viral particle has rapidly evolved in an attempt to evade prophylactic and therapeutic regimens. The phenotypic modifications observed in the VOCs are populated with several S-protein mutations and deletions, as elucidated below.

The B.1.1.7 The alpha variant was first identified in the UK and has since disseminated to more than 110 countries, with the most prominent mutations being the N501Y (role in viral entry into human cells), P681H, H69/V70 and Y144/145 deletions, with the latter two deletions altering the shape of the spike, allowing the virus to evade an immune response^2^. First identified in South Africa, in December 2020, the B.1.351 The Beta variant escalated to one of the most predominant VOCs, harboring the characteristic N501Y, K417N and E484K mutations. The Beta variant has since spread to 68 countries^3^ and shares two common mutations (N501Y and E484K) with the P.1 Gamma variant, first identified in Japan in four travelers from Manaus, Brazil^16^. Since its detection, the Gamma variant has spread to more than 37 countries with signature mutations, including N501Y, K417N, and E484K, which may aid the virus in evading an immune response. The B.1.617 variant appeared in India in October 2020. This variant continued to evolve into B.1.617.1, known as the Kappa variant, and B.1.617.2, known as Delta variant, which is now the most common variant in India, having two prominent mutations, E484Q and L452R. Last, the B.1.427/429 Epsilon lineage appeared for the first time in California and carried the L452R mutation, which has been reported to be highly contagious^17^. Taken together, the mentioned mutations within VOCs are of functional importance, thus augmenting public health concerns surrounding potential detrimental clinical implications, including infection volatility and vaccine efficacy^17–20^. Key VOC mutations may also result in diagnostic test failures and could potentially lead to a decrease in the affinity of antiviral candidates for SARS-CoV-2, thus necessitating the rollout of recurring SARS-CoV-2 vaccines.

In the last year, wastewater surveillance has been used across the globe to monitor for the presence of COVID-19 infections within a community and to track infection trends^21–23^. To date, limited studies have utilized wastewater to routinely screen for circulating variants^11,24–26^. Furthermore, most studies used next-generation sequencing (NGS) to gather information about the diversity of SARS-CoV-2 variants at the community level^27,28^. While NGS sequencing is informative to identify new variants, it is costly to perform routine screening of sewage samples to identify and profile known mutations associated with VOCs. As such, this study aimed to conduct weekly wastewater surveillance using a robust TaqMan SARS-CoV-2 mutation panel and report on key VOCs at the community level within 24 WWTPs in Cape Town, South Africa. This study also identified and determined the emergence of the VOC that drove South Africa’s third wave and elaborated on the key structural implications of VOC mutations on viral infectivity.

## Results and discussion

**Figure 1:**
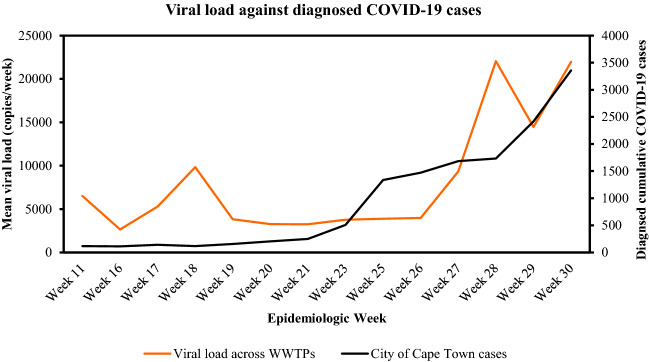
Temporal analysis of SARS-Cov-2 viral load in wastewater against diagnosed COVID-19 cases in Western Cape.

In the current study, routine surveillance was conducted on 272 wastewater samples within 24 WWTPs in the Cape Town Municipality, South Africa. At the start of the experiments, the epidemiological limit of detection (LOD) was determined and set at 700 g.c./mL. The latter results were higher than the LOD of 50 g.c/mL reported by Randazzo et al.^29^. Subsequently, RT-qPCR analysis was used to confirm the presence and quantify the SARS-CoV-2 viral load within the 24 WWTP between April and July 2021. Over the three months, the average viral load was 12 798 g.c./mL, and only sites with a viral load > 1500 g.c/mL were used for subsequent variant testing (Fig. 1).

This study reports on the emergence of a highly fit SARS-CoV-2 lineage with several S-protein mutations that decrease vaccine efficacy while preluding possible reinfection. Wastewater contains a mixture of inactivated SARS-CoV-2 nucleic acid fragments shed from multiple infected individuals. Genotyping these fragments can identify the circulating variant and its geographic distribution within a given catchment area with much less effort than clinical surveillance^30^. This is especially important with the current lineage of SARS-CoV-2 that is constantly mutating, affecting vaccine efficacy and viral transmission. Understanding the dynamics of a highly virulent circulating strain will allow for prompt response and informed decision making to control and restrain viral spread. Likewise, recent scientific evidence highlighted the importance of wastewater surveillance in tracking disease prevalence in the general population and, more recently, tracking signature mutations in VOC. Hence, we report a robust TaqMan SARS-CoV-2 genotyping method to monitor wastewater for signature mutations linked to VOCs at the population level. Additionally, this information will identify spatial hotspots while tracking temporal trends of VOCs, including their prevalence and frequency over time.

As the Western Cape, South Africa approached the third wave (early June 2021 week 24), a steady increase in SARS-CoV-2 viral load was observed, with an average of 12 798 genome copies (g.c.) /mL recorded over a 3-month period. The orange line graph represents the average viral load across all wastewater treatment plant, whilst the black line graph indicates diagnosed cumulative COVID-19 cases within the Western Cape (Fig. 1).

**Figure 2:**
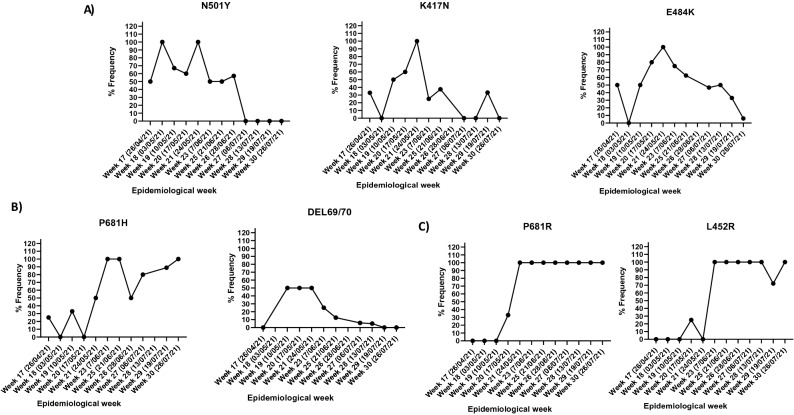
Mutation frequency linked to key Variants of Concern (VOC). Frequency of viral RNA showing spike protein mutation linked to the (**A**) Beta variant with three key substitutions in the RBD (K417N, E484K and N501Y), (**B**) the Alpha variant with the N501Y, P681H and 69/70 Deletion and, (**C**) the Delta variant harbouring mutations for P681R and L452R.

**Figure 3:**
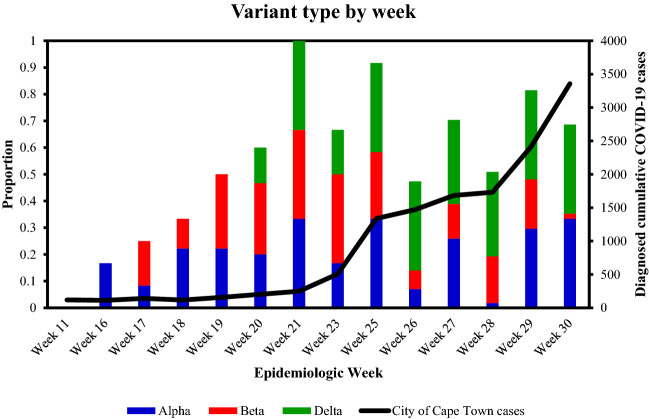
Temporal analysis of SARS-CoV-2 variants of concern compared to diagnosed COVID-19 cases. Data suggests that the Beta variant predominance decreases with dominance of Delta variant. Data is represented as a stacked bar graph. Each colour represents the average frequency of a variant type that occurs across all WWTPs, and the proportion of that specific variant compared to other variants is given by week.

Next, the mutational frequency, which refers to the proportional of wastewater samples that were positive for a mutation divided by the total number of samples, where detected for all VOC. Figure 2 displays the mutational frequency for the Alpha (N501Y, 69/70DEL and P681H), Beta (K417N, N501Y and E484K) and Delta (L452R and P681R) variants of SARS-CoV-2 found in sewage water over a period of 3 months. According to Abdool Karim et al.^6^ and later Tegally et al.^3^, the Beta variant was the predominant circulating variant within the Western Cape. Our results confirmed previous reports showing that the Beta variant was indeed the predominant variant between 26 April 2021 and 24 May 2021 (Epidemiological weeks 17 and 21, respectively, Fig. 3). This study also showed that the Beta variant harbored a combination of key mutations (K417N, N501Y and E484K) that define this lineage at a stable frequency of > 60% (Fig. 2A). The presence of these mutations was also confirmed by whole genome sequencing of a subset of samples collected from selected WWTPs within the Cape town municipalities (Supplementary data). However, the frequency for mutations of the Alpha variant was very low, corresponding to a prevalence ≤ 40%, while the Delta variant was not introduced during the respective period  (Figs. 2B,C and 3). Temporal analysis showed that from 7 June to 26 July 2021, the combination of K417N, N501Y and E484K mutations for the beta variant decreased at a mean frequency of < 40% (Figs. 2 and 3). The surge in viral load and decreased occurrence of various mutations of the Beta variant were followed by a simultaneous rise in the frequency of mutations corresponding to the Delta variant. In the current study, genotyping of SARS-CoV-2 RNA for key mutations of the Delta variant (L452R and P681R) and whole genome sequencing (Supplementary data) confirmed the presence of this variant within wastewater by mid-May 2021, corresponding to epiweek 20 (Figs. 2C and 3).

**Figure 4:**
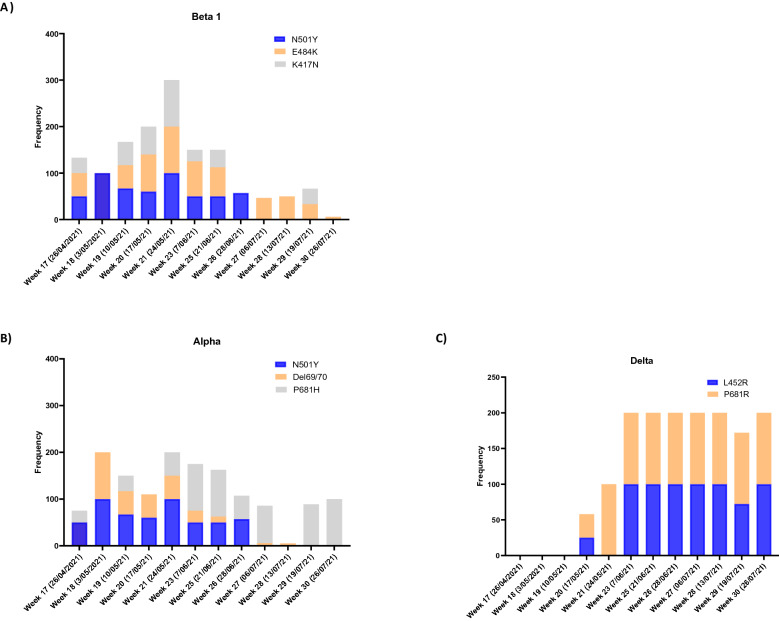
Temporal analysis of the City of Cape Town municipality WWTP. The frequency of the spike protein mutations during the epidemiological weeks seeks to explain patterns of the shift of key mutations linked to 3 variants of concern; (**A**) Beta, (**B**) Alpha, and (**C**) Delta variant detected over time. The mutational frequency on the y axis is represented by a stacked bar graph. Each colour represents the average mutation frequency of a variant type, which refers to the proportion of wastewater samples that were positive for a mutation divided by the total number of samples.

Our data also revealed the cooccurrences of key Delta mutations (L452R and P681R) with the P681H mutation corresponding to the Alpha variant (Fig. 2). Happi et al. (2020) reported that the P681H mutation was first observed in March 2020 in Nigeria and Hawaii, whereafter it was detected in New York and Israel and is now characterized as part of the global spreading B.1.1.7 VOC (35, 36). In this study, the P681H mutation was introduced as early as April, even when the viral load was low (Fig. 1B). An erratic pattern in the frequency of the P681H mutation was observed, with the lowest frequency of 25% across most of the sites. Thereafter, the P681H mutation became static across various sample sites, showing a frequency of 100% throughout the peak of the third wave (Figs. 2B, 3 and 4B). According to Shang et al. (37) and Zukerman et al. (35), the P681H mutation is a critical mutation found in the proximity of the furin cleavage site. Although it has been postulated that the P681H mutations play a role in increased furin cleavage, allowing virus entry into the cell, they do not decrease vaccine efficacy (35, 38, 39). Nonetheless, the P681H mutations are still being monitored globally (35).

Furthermore, apart from the P681H mutation, a 69/70 deletion, a key mutation for the Alpha variant, was observed only in two sample sites, namely, the Mitchells Plain and Zandvliet WWTP. It could be speculated that this variant could have been endemic to those catchment areas but not transmissible, as the viral load remained high throughout May 2021 (frequency > 50%) without offsetting any resurgence (Figs. 2 and  4).

### Delta variant predominant in the third wave

This study evaluated whether the Delta variant was the dominant variant during the third wave, identifying when it emerged and elaborating on the protein structure of key mutations that may affect cellular infectivity. The Delta variant was first detected in India and became the leading variant globally^31–33^. The Delta variant is 60% more transmissible than its ancestry SARS-CoV-2 strain, meaning infection is more likely to occur in those exposed to the strain^34^.

**Figure 5:**
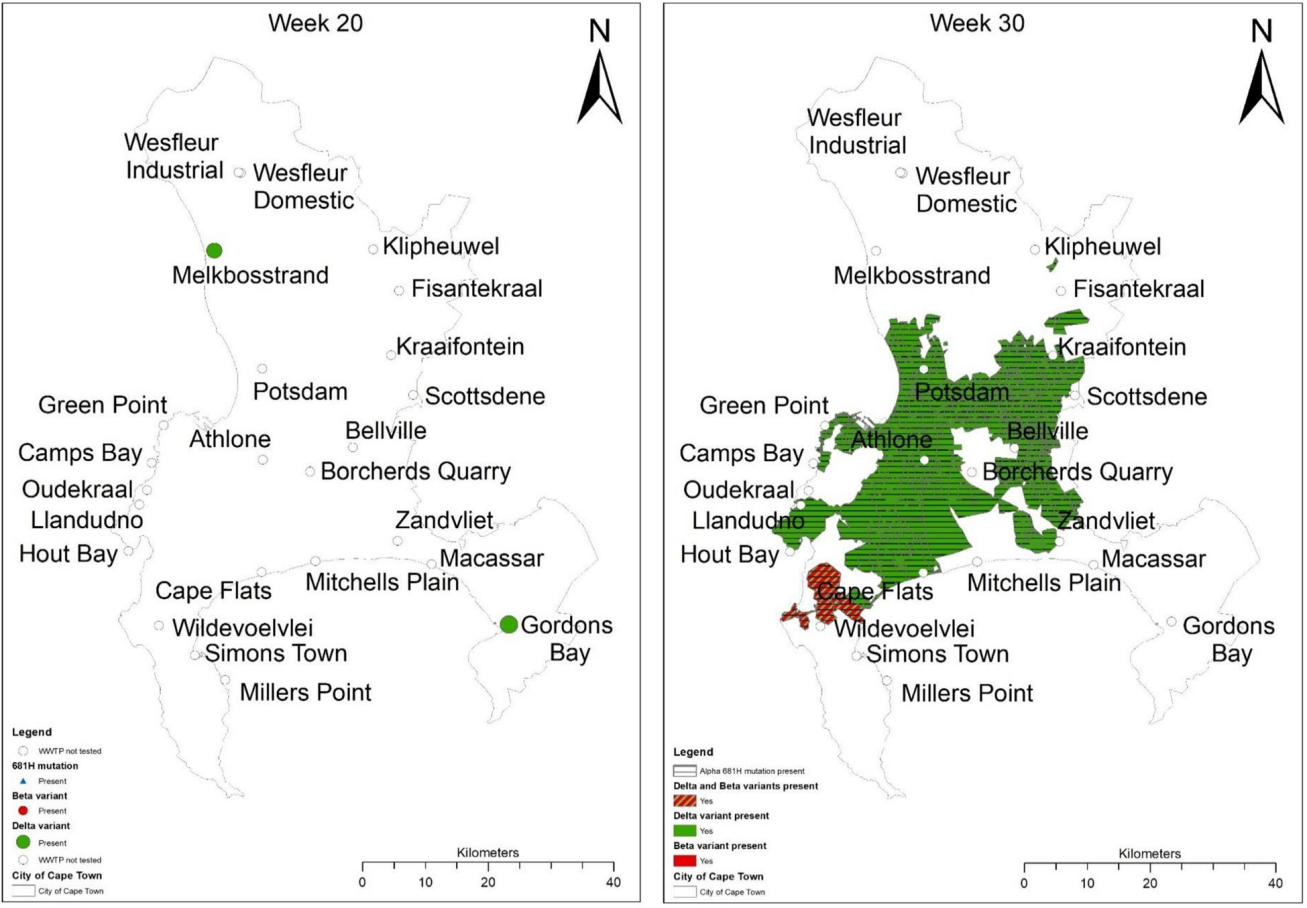
Spatial analysis of the rapidly evolving Delta variant over three months. The Delta variant was initially detected in the Melkbosstrand WWTP, where after it spread to the remaining catchment areas. Spatial mapping showed that the Delta variant was detected in more than 95% of the WWTP. Red-Beta variant, Green-Delta and Black indicates the presence of the P681H mutation. All maps were produced using ArcGIS 10.6.1 (https://www.arcgis.com/).

The spatiotemporal analysis showed that the Delta variant was initially detected in the Gordons Bay and Melkbosstrand WWTP, whereafter it spread to the remaining 15 catchment areas within the City of Cape Town (Figs. 4 and 5). Figures 3 and 4 confirmed that as the Western Cape approached the third wave (early June 2021), a steady increase in SARS-CoV-2 viral load was observed, which was concomitant with the observed increase in the Delta variant. Spatial mapping showed that between 6 June and 21 July 2021, the Delta variant was detected in more than 95% of the WWTPs with a viral load ranging between 2000 and 50 000 g.c/mL (Figs. 3, 4 and 5). This co-occurred with a decline in the Beta and Alpha variants (Figs. 3 and 4), suggesting that this heightened prevalence observed during the third wave could have been attributed to the circulating Delta variant. Similar findings were reported by Scheepers et al. (2021), with the Delta variant being predominant by the end of June (317/422, 75% in week 24; 203/230, 88% in week 25), with the first mutations being identified within the Western Cape in Epidemiological 21^10^. The latter finding confirmed our study results; however, using wastewater, the Delta variant was detected as early as Epidemiological week 20 (Fig. 5). Furthermore, it can be speculated that the rapid emergence and spread of the Delta variant could have been introduced during travel-related importations, whereafter it continued to spread within the population, resulting in possible community transmission. However, evidence related to community transmission remains inconclusive. Wastewater surveillance and the use thereof to track, discriminate, and quantitate SARS-CoV-2 variants using targeted surveillance, such as NGS sequencing and the highly specific and discriminatory VOC RT-qPCR mutation panel, has become a commonly adopted method. However, despite allowing for the fast and cost-effective detection of signature mutations linked to VOC, it should be noted that wastewater samples contain a mixture of different isolates. Therefore, the method can only be utilized to detect the circulating variant in the community and cannot discriminate that the mutations linked to the VOC are present on the same virus genome.

Nonetheless, collectively, our results suggest that wastewater can be used as a proxy for prompt detection and informed decision making that will allow health officials to introduce rapid nonpharmaceutical interventions to curb the spread of this highly transmissible strain.

### Structural Insights into SARS-CoV-2 Spike Glycoprotein VOCs on Cellular Infectivity

To further elucidate the understanding and consequences of these VOCs within the SARS-CoV-2 spike protein, in silico modeling techniques were utilized. This was done by comparing the wild-type versus mutant RBD domain when bound to the ACE2 receptor, as well as the affinity of the furin enzyme to the S1/S2 boundary site when mutated.

### Infectivity

**Figure 6:**
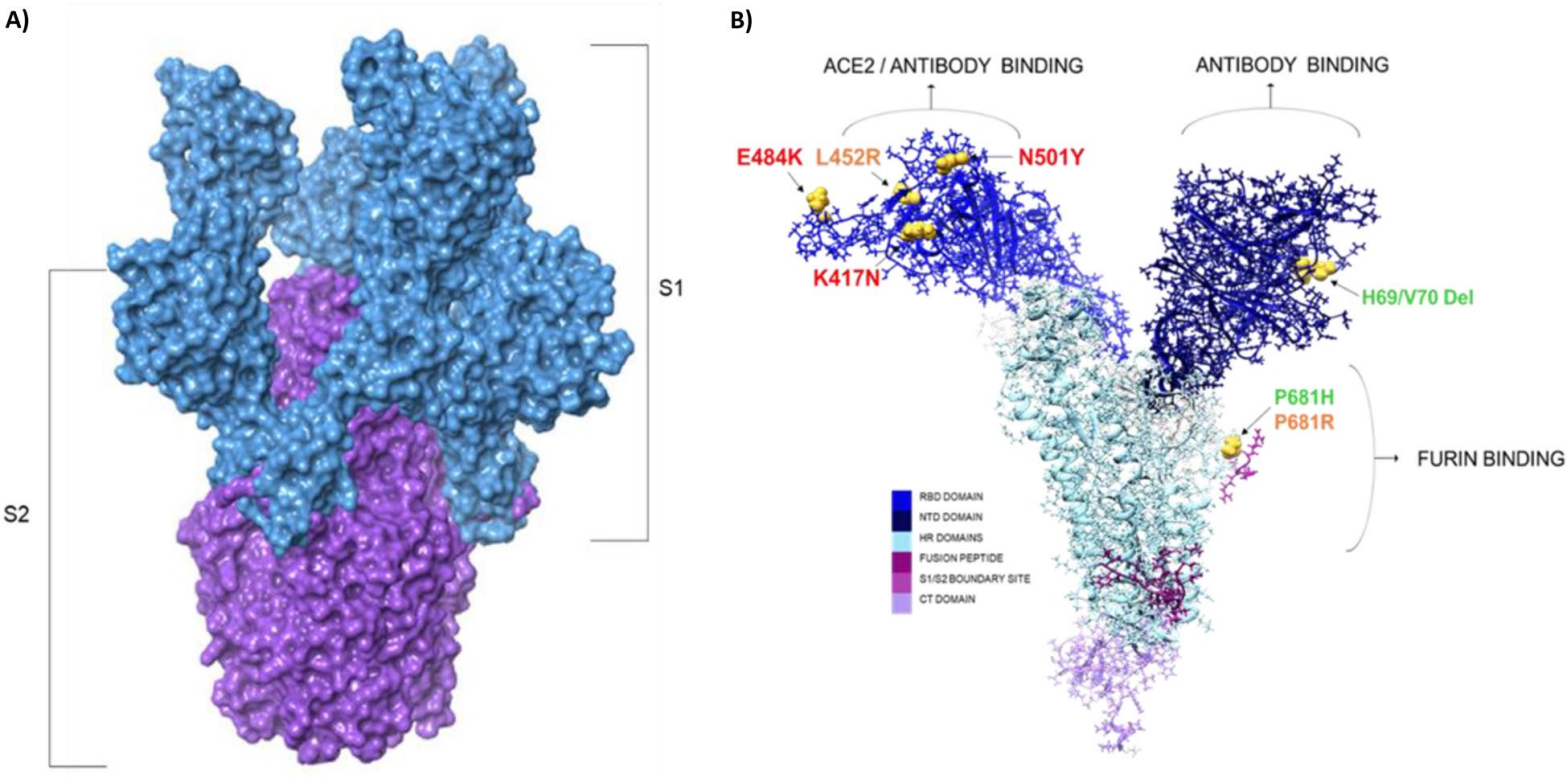
Schematic representation of SARS-CoV-2 spike glycoprotein: Schematic representation of SARS-CoV-2 spike glycoprotein: (**A**) Surface area representation of trimeric spike protein conferring an “open conformation” RBD domain (S1 domain depicted in blue and the S2 domain represented in purple) (**B**) Atomistic representation of characterized subdomains within the monomeric spike protein and the spatial mapping of defined protein binding regions. The identified VOCs within the RBD, NTD and S1/S2 boundary site are also demonstrated. The Alpha variant mutation and deletion are represented in green, the Beta variant in red and the Delta variant in orange.

Binding of the spike protein RBD to ACE2 is critical to viral replication. After RBD-ACE2 binding, furin binds to the S1/S2 boundary site; thus, mutations seen at both of these domains can have detrimental implications on patients who lack neutralizing antibodies. Numerous studies have eluded the chemical inferences of mutations within the spike protein, with most in silico studies concluding that amino acid alterations, specifically at the RBD domain, increase ACE2-spike interactions (2,5–7). This study chose to assess two vital binding regions of the spike protein by comparing mutants from four of the major VOCs assessed in wastewater RT-QPCR surveillance. The mutations/deletions of concern were computed as per VOC, followed by molecular docking and interaction analysis to identify and compare the binding landscape between the viral and host proteins. Table 1 demonstrates the wild-type (WT) and VOCs complexed with ACE2 and Furin (Fig. 6).

**Table 1:** Summary of docked ACE2 and furin protein to SARS-CoV-2 spike WT and VOCs.

Complex	Docking score	Number of Interfacing Residues (Spike VOC/Protein)	Interface Area (Å^2^) (Spike VOC/Protein)	Number of Salt Bridges	No of Hydrogen Bonds	Number of nonbonded interactions
WT-ACE2	8302	17/18	868/887	1	3	161
Beta-ACE2	10,296	24/21	1167/1231	1	7	216
Alpha-ACE2	8378	26/20	1078/1188	1	2	298
Delta-ACE2	9816	23/19	1086/1068	1	3	309
WT-Furin	8210	39/36	1748/1660	1	11	825
Beta-Furin	8210	15/21	1175/1077	1	2	147
Alpha-Furin	7080	24/24	1233/1249	1	5	342
Delta-Furin	10,898	41/42	2007/1897	3	11	748

The results are represented as Patchdock docking scores and PDBsum binding interaction analysis of the binding landscape.

It was evident from the docked complexes that direct binding site mutations did not alter the binding affinity of either ACE2 or furin (Table 1). Increased binding affinity was noted when ACE2 bound to the Delta variant compared to the WT. This was evidenced by an increase of 7 interacting residues and a surface interaction area of 399 Å2. The alpha variant docked to ACE2 with a suboptimal binding affinity. However, interaction analysis revealed 4 additional binding site residues, which led to an increased surface interaction area of 77 Å^2^ when compared to the Delta complex.

The Beta-ACE2 complex demonstrated the best docked structure, indicative of the increased interaction surface area of both proteins. The alteration from LYS to ASN at amino acid 417 resulted in the formation of a hydrogen bond with LYS31 of ACE2 (Supplementary). Compared to the other VOC docked structures, 4 additional hydrogen bonds and approximately double the hydrophobic interactions were noted. Interestingly, these common functional amino acids identified in the spike and ACE2 proteins have previously been reported to upregulate spike-ACE2 binding^35^. It is evident that Beta variant mutations induce stabilizing intermolecular forces, thus increasing the affinity of ACE2 to the altered RBD and potentially increasing infectivity.

An additional integral viral entry-point complex was assessed to further rationalize the increased dissemination of the viral mutants. Furin was docked to the “PRRA” S1/S2 boundary site of the spike protein. Docking results indicated the most favorable binding affinity between furin and the Delta variant spike protein, characterized by a P681R alteration at the S1/S2 boundary site. The Beta variant and WT proteins demonstrated identical docking scores, while the Alpha-Furin complex displayed the least optimal binding affinity. A similar trend was noted in the interaction analysis, with the Beta-Furin complex resulting in the lowest number of intermolecular interactions. The Delta-Furin complex constituted 41 interacting residues, with an interaction surface area of 2007 Å^2^. Of particular interest to the infectivity rate observed with the Delta variant is forming the triple ARG cleavage site. The positively charged ARG mutation at residue 681 resulted in a salt bridge with the negatively charged ASP191 of furin. This attractive force and the increased overall positive charge would increase the affinity of furin to the spike protein. This results in a unique Delta variant, as this additional salt bridge is reliant on the mutation. A characteristic feature of the PRO amino acid is the lack of a hydrogen located on the alpha-amino group. This hinders the amino acid from protonating secondary structures of the protein, thus resulting in the formation of protein loops or turns^25^. The HIS amino acid is positively charged and is usually characterized by its ability to allow for protein folding, stability and function^36^. As a result of the above information, it is not surprising that the alpha-furin complex contained the least optimal binding landscape. The PRO-HIS alteration at amino acid 681 could lead to a more stable protein structure at the S1/S2 binding site, resulting in “energy-heavy” furin cleavage.

For this study, only molecular docking was assessed to confer a general understanding of the structural mechanics. To gain a more comprehensive and reliable dataset, molecular dynamic simulations are required. This will evaluate the reactivity of all the mutations in a VOC by allowing for flexibility within the complex. This is especially important when elucidating the consequences of the Alpha variant H69/V70 deletion on structural loop dynamics and spike protein stability.

It is evident from the binding of ACE2 and furin to the spike VOCs that the Delta variant results in increased binding of both host proteins and thus suggests a possible molecular mechanism of the current excessive infectivity rates.

## Materials and methods

### Wastewater sample concentration and RNA extraction

Raw sewage samples were collected weekly from 24 WWTPs in Cape Town, South Africa, between 19 May-31 July 2021 (16 weeks). For RNA extraction, 100 mL of grab sample was mixed and centrifuged at 2500 xg for 20 min, whereafter 2.5 mL of the resultant pellet was used for total RNA extraction using a previously described protocol of Johnson et al. (2021). Total RNA was subjected to spectrophotometry using the NanoDrop ND-8000 (Thermo Scientific, USA). Only samples with a A260/280 ratio between 1.8–2 and a A260/230 ratio between 1.8–2.1 was used for subsequent After isolation, the consequent RNA (70 µL) was aliquoted and stored at –80 °C until required for molecular analysis.

### Detection of spiked inactivated SARS-CoV-2 in wastewater sample as a positive extraction control

Inactivated SARS-CoV-2 was cultured from a nasopharyngeal swab for both wild type (E6.2) and variant (501Y. V2) was previously isolated and fully characterized and was a kind gift from Prof Wolfgang Preiser (National Health Laboratory Services, University of Stellenbosch, Tygerberg, Cape Town)^3^. The viral titer of inactivated SARS-CoV-2 was calculated and found to be 1 × 10^6^ g.c./µL. Wastewater matrix samples, which previously were negative for SARS-CoV-2, were spiked with and without the inactivated wild-type and mutant virus at a concentration of 2 × 10^4^ g.c/mL. RNA was extracted and used as an extraction positive control in all subsequent real-time quantitative polymerase chain reactions (RT-qPCR) reactions.

### Real-time quantitative polymerase chain reaction (RT-qPCR) analysis

For quality assurance and control (QC/QA) the combined primer/probe mix (Integrated DNA Technologies, Coralville, IA, USA) (Table 2) as well as PCR positive controls were resuspended, aliquot and stored at − 20 ºC to limit freeze thaw cycles. For each PCR run, (i) a cultured inactivated nasopharyngeal swab SARS-CoV-2 spike extraction positive control, (ii) a extraction negative control (wastewater sample with absence of SARS-CoV-2), (iii) a PCR non-template control (NTC; nuclease free water, Cat no. 11–04-02–01) (Integrated DNA Technologies, Coralville, IA, USA), as well as a (iv) Synthetic SARS-CoV-2 DNA plasmid at 200 g.c/µL (2019-nCoV_N Positive; Cat no. 10006625) (Integrated DNA Technologies, Coralville, IA, USA) was included in each PCR run. As per our in-house SOP, failure of any one of these controls invalidated the test.

**Table 2:** Thermal cycling conditions, primers/probes for RT-qPCR and SNP genotyping used in the study.

Organism	Assay Cat number	Company	Target	Sequence	Cycling parameters
**Real-time quantitative polymerase chain reaction (RT-qPCR) analysis**					
SARS-CoV-2	2019-nCoV CDC EUA kit (Cat number RV202001)	Integrated DNA Technologies, Coralville, IA, USA	N1	F 5′-GAC CCC AAA ATC AGC GAA AT-3′ R 5′-TCT GGT TAC TGC CAG TTG AAT CTG-3′ P-FAM-ACC CCG CAT TAC GTT TGG TGG ACC-BHQ1	Reverse transcription and RT-qPCR reaction: 50º for 10 min; 95º for 3 min Amplification: 95º for 15 s; 60º for 1 min (40 cycles)
	2019-nCoV CDC EUA kit (Cat number RV202002)		N2	F 5′-TTA CAA ACA TTG GCC GCA AA-3′ R 5′-GCG CGA CAT TCC GAA GAA-3′ P-FAM-ACA ATT TGC CCC CAG CGC TTC AG-BHQ1	

SNP genotyping	Assay Cat number	Company	Target	TaqMan SARS-CoV-2 mutational Panel	Cycling parameters
**Genotyping analysis: TaqMan SARS-CoV-2 mutational Panel**					
SARS-CoV-2	A15300	Thermo Fisher Scientific, Waltham, USA	Spike protein	Taq path Master Mix 10 mL	Pre-read 60º for 30 s; Reverse Transcription 50º for 10 min; Fast DNA polymerase activation 95º for 2 min; PCR(45 cycles) 95º for 3 s 60º for 30 s Post-read 60º for 30 s
	A51808			S.delH69V70	
	A51814			S.K417N.AAG.AAT	
	A51813			S.E484K.GAA.AAA	
	A51816			S.P681H.CCT.CAT	
	A51822			S.P681R.CCT.CGT	
	A51819			S.L452R.CTG.CGG	
	A51812			S.N501Y.AAT.TAT	

For all PCR reactions, the Minimum Information for Publication of Quantitative Real-Time PCR experiments (MIQC) guidelines was followed to ensure reliability and data integrity^37^. For each run, the following RT-qPCR MIQC detail was adhere to; (i) the threshold was set at 0.02 which was in the linear phase of the amplification plot, (ii) PCR efficiency was considered acceptable if in the range of 90–100%, (iii) the slope was acceptable if between − 3.55 and − 3.30, (iv) all R^2^ values was acceptable if > 0.99, (v) a minimum of 5 points for the dilution series was used to generate the standard curve and (vi) all samples was run in duplicate and a minimum SD range of < 0.5 was required for both standards and samples. Next, the presence of SARS-CoV-2 was determined and quantified using the Centre for Disease Control and Prevention (CDC) approved 2019-nCoV CDC EUA Combined primer/probe kit (Cat no. 10006670) (Integrated DNA Technologies, Coralville, IA, USA) using N1 and N2 as a target (Table 2). For quantitative analysis, the standard curve method was employed using the 2019-nCoV_N Positive Control (Cat no. 10006625) (Integrated DNA Technologies, Coralville, IA, USA) to construct the serial diluted calibrator/ standard curve matric of 200 000- 20 g.c./µL , whilst the threshold was set in the linear phase at 0.02 to ensure that all samples amplified above the threshold with a correlation coeffient > 0.99 and a PCR efficiency between 90–100% (slope − 3.30 to − 3.55). SARS-CoV-2 viral RNA from both sewage and spiked control samples was reversed, transcribed and quantified using the Bio-Rad iTaq Universal Probes One-Step Kit (Cat no. 1725141, Bio-Rad Laboratories, Hercules, CA) according to the manufacturer’s instructions. Extracted RNA was standardized at a concentration of 0.2 µg/µL, and RT-qPCR was performed as previously described by Johnson et al.^38^. Briefly, 1 µL of 0.2 µg/mL RNA was added to 2.5 µL iTaq Universal Probe One Step mix (Bio-Rad, Hercules, CA), in a final volume of 10 µL containing 0.5 µL of the IDT 2019-nCoV CDC approved nucleocapsid (N) primer/probe set (Cat no. RV202001/2, Integrated DNA Technologies, Coralville, IA, USA) and 3.25 µL nuclease free water (Cat no. 11-04-02-01, Integrated DNA Technologies, Coralville, IA, USA). PCR was conducted on an Applied Biosystems™ QuantStudio™ 7 Flex Real-Time PCR System (Thermo Fisher Scientific, Waltham, USA) under the following cycling conditions: Reverse transcription and RT-qPCR reaction was done at 50° for 10 min; followed by a 95° for 3 min and amplification was done at 95° for 15 s and 60° for 1 min (40 cycles) (Table 2). All reactions were performed in duplicate, and a plasmid SARS-CoV-2 positive control at a viral titer of 200 g.c./µL was included as a RT-qPCR positive control.

All Subsequently, samples positive for SARS-CoV-2 were used to detect S-protein signature mutations for the Alpha (N501Y, DelH69V70 and P681H)^39^, Beta (N501Y, E484K, and K417N)^3^ and Delta (L452R, and P681R)^40^ variants using the TaqMan SARS-CoV-2 Mutation Panel as per manufacturer’s instructions (Thermo Fisher Scientific, Waltham, USA) (Table 2).

### Calibration curves and limit of detection determination

Given the complexity of the wastewater matrix, the detection limit was used to determine the lowest viral load the assay will detect in g.c./µL. Briefly, a tenfold serial dilution from 200 000–1 g.c./µL of RNA was run in duplicate using the 2019-nCoV_N plasmid control as the calibrator as well as the spike wastewater sample to confirm the limit of detection (LOD) using the N-gene. Linear regression was performed between the log copy number and the Ct values from the RT-qPCR results on all calibration curves that resulted in a good fit and allowed for the determination of the detection limit.

### Genotyping analysis to detect mutations within Variant of Concern (VOCs) in wastewater

To perform variant detection, allele-specific RT-qPCR was performed in duplicate using the TaqPath™ RT-qPCR assay (Table 2, Thermo Fisher Scientific, Waltham, USA) according to the manufacturer’s instructions. Briefly, a SNP genotyping reaction was set up in duplicate with the addition of 4 µL of RNA with a viral titer > 1500 g.c./mL, 2.5 µL of TaqPath one-step RT-qPCR master mix and 0.25 µL of TaqMan™ SARS-CoV-2 Mutation Panel (Table 2) and this reaction was made up with nuclease free water to a final volume of 10 µL. The following variants of interest were tested for each assay: Alpha, Beta, and Delta with mutations N501Y (Cat no. A51812), E484K (Cat no. A51813), K417N (Cat no. A51814), DelH69V70 (Cat no.: A51808), P681H (Cat no. A51816), L452R (Cat no. A51819), and P681R (Cat no. A51822) (Table 2).

Next, the Genotyping PCR was conducted as specified in Table 2. Briefly, PCR cycling was performed on an Applied Biosystems™ QuantStudio™ 7 Flex Real-Time PCR System instrument (Thermo Fisher Scientific, Waltham, USA) using the following conditions: reverse transcription was performed at 60 °C for 30 s followed by 50 °C for 10 min, Taq polymerase activation at 95 °C for 2 min, followed by 45 cycles of denaturation at 95 °C for 3 s, and annealing of 30 s at 60 °C with a final Postread for 30 s at 60 °C. All RT-qPCR experiments included a NTC, a wild-type AcroMetrix Coronavirus 2019 (COVID-19) RNA control (RUO) (Cat no. 954519, (Thermo Fisher Scientific, Waltham, USA) supplied with the assay, and the extracted nucleic acids from wild-type and variant nasopharyngeal swabs positive for SARS-CoV-2 PCR.

### Whole-genome sequencing and genome assembly

#### Library preparation and sequencing

SARS-CoV-2 RNA libraries were produced using the ATOPlex platform, which is a multiplexed amplicon-based sequencing workflow that is tailor-made for detecting SARS-CoV-2 fragments (~ 500 bp). Following reverse transcription of 10 µL RNA, a two-step PCR with 13 and 27 cycles for target enrichment and sequence adapter with dual-barcode primer (Cat No: 1000021626) addition, respectively, was carried out in the first and second PCRs. The amplified PCR products were purified by magnetic bead-based techniques followed by quantity and quality checks using the Qubit dsDNA high sensitivity assay kit (Life Technologies, USA) and gel-based electrophoreses, respectively. Libraries were equally pooled, and single strand circularized DNA (ssDNA) was synthesized using the MGIEasy Dual Barcode Circularization kit (Cat No:1000020570). DNA nanoballs (DNBs) were constructed for both the ssDNA sample library and a dual barcoded balance library (Cat No: 1000022270) in parallel, which was then mixed 3:1 prior to sequencing on the DNBSEQ-G50RS instrument at SAMRC’s Genomics Centre. Data analysis was conducted using a sweet of bioinformatics tools (https://github.com/MGI-tech-bioinformatics/SARS-CoV-2_Multi-PCR_v1.0).

### In silico characterization of the binding landscape of mutations found within VOCs of the SARS-CoV-2 spike protein

Most available SARS-CoV-2 spike glycoprotein crystal structures either lack the resolved structure of the S1/S2 boundary site or demonstrate an already cleaved spike protein. To assess the binding landscape of the spike protein, all fully formed quaternary structures of the protein must be available. To accommodate this requirement, the S1/S2 boundary site and any other missing residues were homology modeled using the SwissModel webserver^41^. The monomeric model was established from template structures including PDB codes: 7mkm, 6wpt and 7a94. Once modeled, all mutations within the VOCs were computed using UCSF Chimera visualization software and structurally minimized using the AMBER20 molecular dynamics package^42^. Host proteins furin and ACE2 were retrieved from the Protein Data Bank using the PDB codes 4z2a and 7a94, respectively. The proteins were then prepared on UCSF Chimera, in which all nonstandard residues were deleted, hydrogen atoms were added and proteins charged. Patchdock^43^ and Firedock^44^ online webservers were utilized to assess docking at the spike protein. These tools utilize rigid-body docking algorithms to determine the geometric fit within the binding region of the target molecule. This is assessed according to the surface shape of the target molecule. To identify the best docking pose of the VOC complexes, the protein structures were uploaded to the server, and the identified binding regions were defined. Once scored, the best docked complexes were subjected to the firedock webserver to refine and filter the best structures. The best 10 structures were assessed individually on UCSF Chimera software to disregard any complexes that yielded atomistic clashes or steric hindrance. The final structures were then ranked based on docking scores, and the best complex was chosen for further analysis. The identified complexes were then subjected to interaction analysis using the EMBL-EBI PDBsum online webserver^26^. This server provides critical information on the 3D structure of a complex, including the secondary structure, molecules within the complex and schematic diagrams of the interactions within the complex. Intermolecular interactions identified between amino acids in the complex, including salt bridges, hydrogen bonds and other noncontact interactions, are also described. The information provided in PDBsum was subsequently utilized to elucidate the potential molecular mechanisms of increased VOC infectivity.

### Statistical analysis

Descriptive statistics for continuous variable days were calculated with the mean, standard deviation, median, and interquartile range.

### Spatial data

Suburb shapefiles were obtained from the City of Cape Town open data portal. Coordinates for each WWTP were collected using a handheld GPS and verified using Google Earth. All maps were produced using ArcGIS 10.6.1. The WWTP catchment areas (comprising 753 suburbs) were joined to the corresponding WWTPs.

## Conclusion

It is imperative in the battle against COVID-19 to identify hotspots and monitor circulating strain in real time, allowing mitigation strategies to curb further spread of a highly transmissible strain. PCR genotyping provides real-time tracking and detection of circulating variants. This study acknowledges that RT-qPCR genotyping has various limitations compared to the gold standard NGS sequencing that can detect both known and unknown variants. However, PCR genotyping can be routinely performed; it allows for real-time monitoring of VOCs, the technology is readily available in most laboratories, and more importantly, it can provide information on the circulating variant at the population level. Therefore, early detection through WBE can give public health officials an additional and much needed metric to rapidly triangulate the prevalence of circulating variants within a community, making this type of surveillance essential for current and future outbreaks.

## Supplementary Information

Supplementary Figures.

Supplementary Table S1.
