# Supplementary material for: Tracking the circulating SARS-CoV-2 variant of concern in South Africa using wastewater-based epidemiology
Source: Sci Rep. 2022 Jan 21;12:1182. doi: 10.1038/s41598-022-05110-4 (PMC8783013; doi:10.1038/s41598-022-05110-4)
Supplement: Supplementary file 1 — Supplementary Figures. [file 41598_2022_5110_MOESM1_ESM.docx]

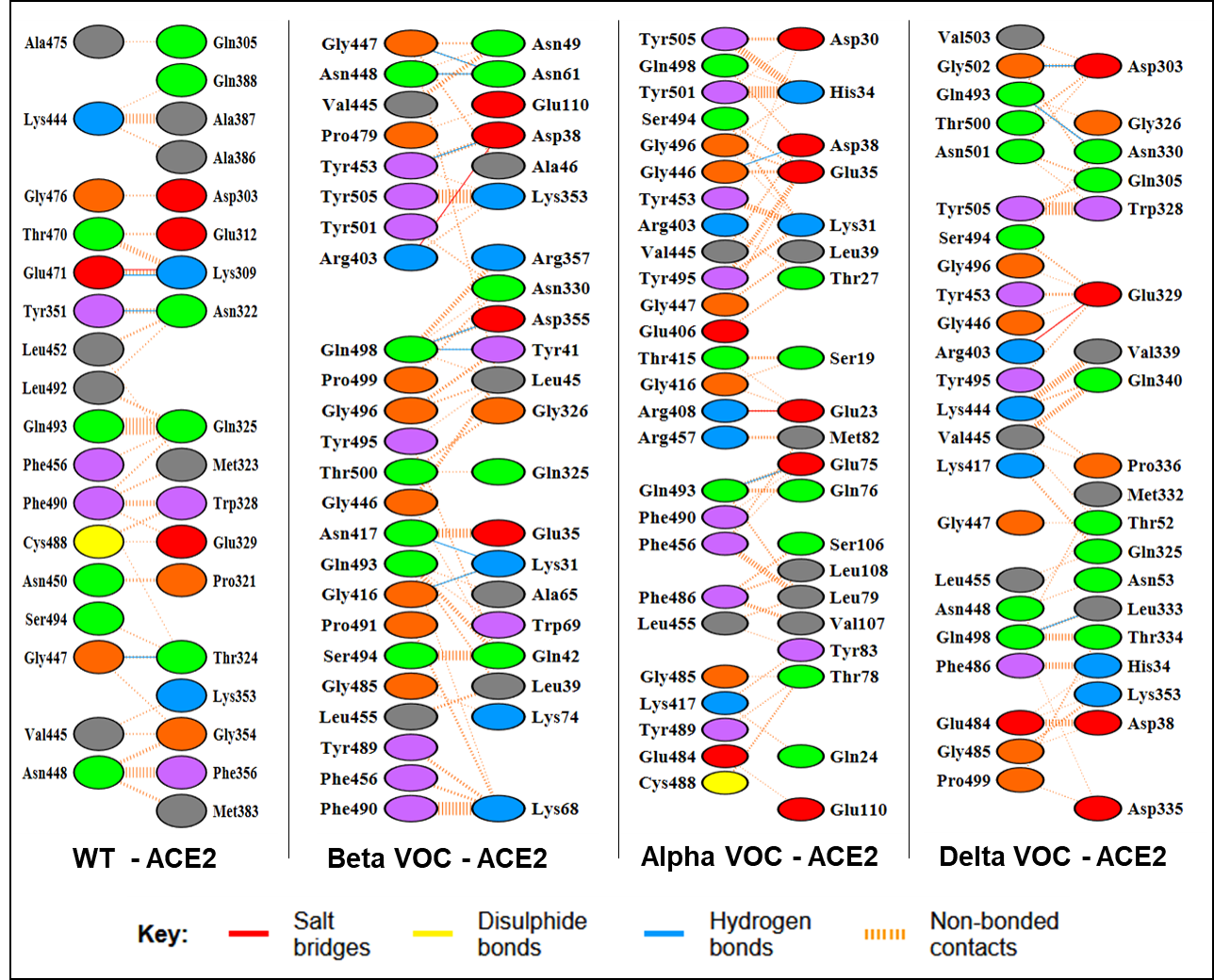


**Figure S1:** Residue interaction plot of ACE2 bound to the RBD domain of the wild-type, Beta, alpha and delta VOC-mutated spike glycoproteins.


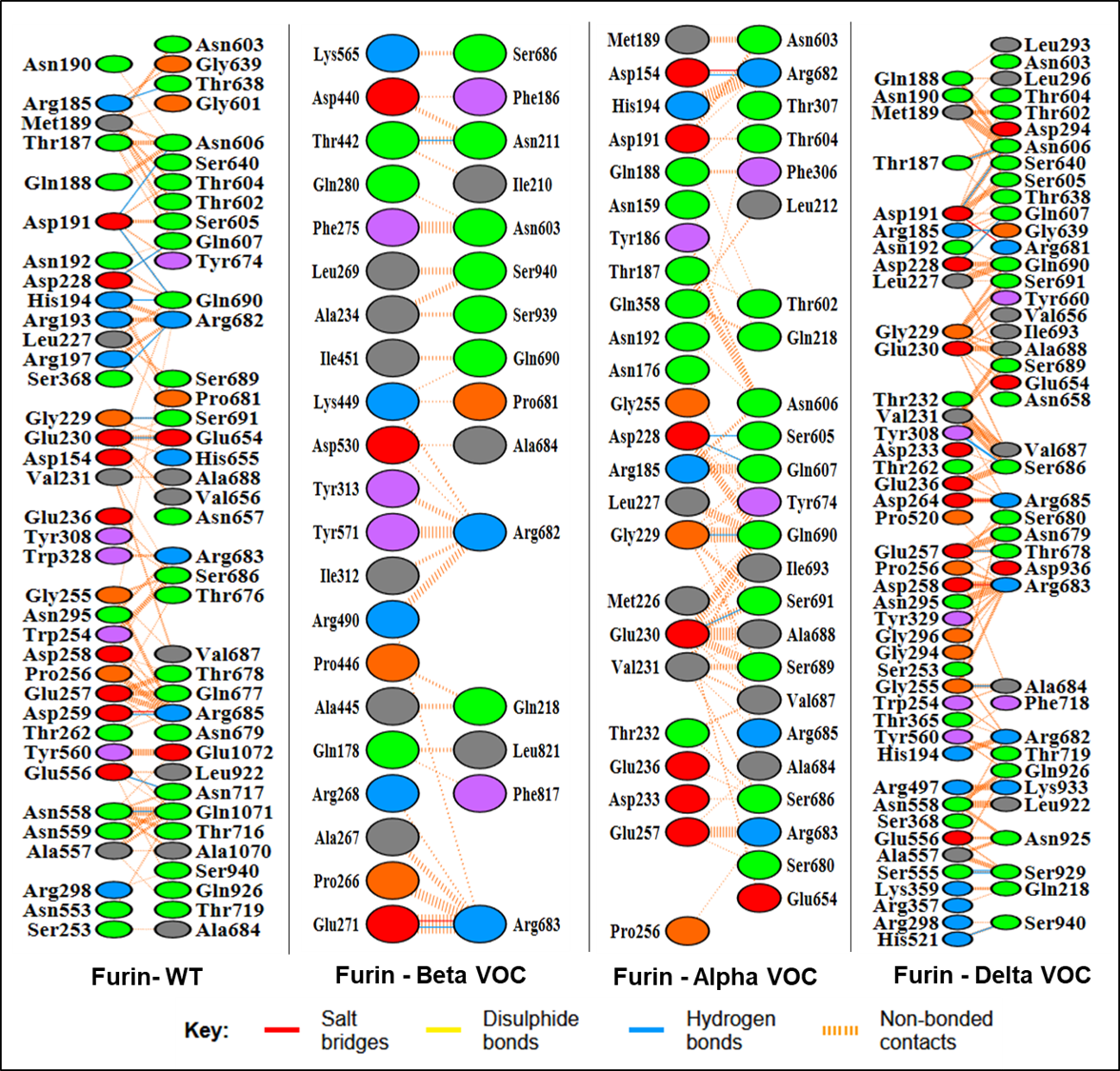


**Figure S2:** Residue interaction plot of furin bound to the S1/S2 boundary site of the wild-type, Beta, alpha and delta VOC-mutated spike glycoproteins.
