# Supplementary material for: Tracking the circulating SARS-CoV-2 variant of concern in South Africa using wastewater-based epidemiology
Source: Sci Rep. 2022 Jan 21;12:1182. doi: 10.1038/s41598-022-05110-4 (PMC8783013; doi:10.1038/s41598-022-05110-4)
Supplement: Supplementary file 2 — Supplementary Table S1. [file 41598_2022_5110_MOESM2_ESM.docx]

**Table S1**: Arithmetic and geometric viral load (copies/mL) for all WWTPs with associated 95% confidence intervals

| **Date** | **Viral load arithmetic mean** | **95% confidence interval** | | **Viral load geometric mean** | **95% confidence interval** | |
| --- | --- | --- | --- | --- | --- | --- |
| Week 16 | 2650 | 1722 | 3578 | 2649 | 1867 | 3760 |
| Week 17 | 5285 | -6337 | 16907 | 2856 | 439 | 18602 |
| Week 18 | 9829 | -21647 | 41304 | 5346 | 201 | 142478 |
| Week 19 | 3828 | -1215 | 8871 | 2490 | 969 | 6399 |
| Week 20 | 3267 | -51896 | 120510 | 6347 | 610 | 66039 |
| Week 21 | 3243 | -14571 | 21057 | 2924 | 8 | 1046007 |
| Week 23 | 3774 | 1205 | 6342 | 3531 | 1818 | 6859 |
| Week 25 | 3880 | 1970 | 5791 | 3429 | 2243 | 5241 |
| Week 26 | 3986 | 2957 | 5016 | 3518 | 2752 | 4496 |
| Week 27 | 9336 | 4435 | 14236 | 6535 | 4348 | 9824 |
| Week 28 | 22051 | 4115 | 39987 | 13170 | 8524 | 20347 |
| Week 29 | 14471 | 9579 | 19362 | 11781 | 8455 | 16415 |
| Week 30 | 21966 | 11724 | 32208 | 16020 | 10391 | 24699 |
